# Quality of Life and Its Psychosocial Determinants Among Cancer Patients: Insights from the Qassim Region, Saudi Arabia

**DOI:** 10.3390/healthcare14070892

**Published:** 2026-03-31

**Authors:** Abdullah Alqifari, Lama Alharbi, Meshari Aloufi, Alwaleed Alsaawi, Fatimah Almutairi, Norah Alodhaybi, Hana Alqifari, Bader Alshamsan

**Affiliations:** 1 Department of Psychiatry, College of Medicine, Qassim University, Buraydah 52571, Qassim, Saudi Arabia; 2 College of Medicine, Qassim University, Buraydah 52581, Qassim, Saudi Arabia; 3 Department of Family Medicine, Buraidah Central Hospital, Qassim Health Cluster, Buraydah 52361, Qassim, Saudi Arabia; 4 Department of Radiology, King Saud Medical City, Riyadh 11196, Saudi Arabia; 5 Oncology Nursing Department, Prince Faisal Cancer Center, Qassim Health Cluster, Buraydah 51911, Qassim, Saudi Arabia; 6 Department of Statistics and Operations Research, College of Science, Qassim University, Buraydah 52571, Qassim, Saudi Arabia; 7 Department of Medicine, College of Medicine, Qassim University, Buraydah 52571, Qassim, Saudi Arabia; 8 Medical Oncology Department, Prince Faisal Cancer Center, Qassim Health Cluster, Buraydah 52366, Qassim, Saudi Arabia

**Keywords:** quality of life, cancer, Qassim, Saudi Arabia, anxiety, depression, psychological support

## Abstract

**Highlights:**

**What are the main findings?**

- Cancer patients in the Qassim region reported a mean global quality-of-life score of 73.8, which is higher than international cancer reference values, despite significant local reporting of physical symptom burden.
- Psychological distress is prevalent, with 29.9% of patients screening positive for depression and 12.8% for anxiety, both of which are significantly associated with poorer global quality of life.

**What are the implications of the main findings?**

- The findings underscore the necessity of integrating routine mental health screening and psychosocial support into standard oncology protocols to address the high prevalence of depression and anxiety.
- Clinical care models in the region should adopt a biopsychosocial approach that prioritizes the management of severe physical symptoms, such as fatigue and pain, to improve overall patient functional status.

**Abstract:**

**Background:** Cancer incidence is increasing in Saudi Arabia and the Qassim region. While survival has improved, quality of life (QoL) remains a vital outcome. However, limited research in Qassim has examined determinants of QoL. This study assessed factors associated with QoL among cancer patients, focusing on sociodemographic, clinical, and psychological variables. **Methods:** A cross-sectional study was conducted at the Oncology Centre in Qassim (January–June 2024). QoL was measured using the Arabic version of the EORTC QLQ-C30, and psychological distress was assessed using the GAD-7 and PHQ-9. Statistical analyses were performed using SPSS v30. Chi-square and Mann–Whitney U tests were used to compare group differences, and logistic regression was used to identify factors associated with global QoL. **Results:** Among 187 patients, the median age was 48 years (range, 18–94), and 73.8% were female. Breast cancer (52.2%) and colorectal cancer (16.1%) were the most common diagnoses. The mean global quality-of-life score was 73.8 (*SD* = 22.2). Anxiety and depression prevalence rates were 12.8% and 29.9%, respectively. Moreover, psychiatric history and psychological distress were significantly associated with poorer QoL in unadjusted analyses. Only higher depression scores were independently associated with lower odds of good QoL (AOR = 0.85, 95% CI [0.77–0.95], *p* = 0.004). Physical and emotional functioning were significantly higher among patients with good global QoL (both *p* < 0.001). In contrast, fatigue, pain, nausea and vomiting, and insomnia were significantly more common among those with poor QoL (*p* < 0.001, *p* < 0.001, *p* = 0.005, and *p* < 0.001, respectively). Based on Therapeutic Change Index thresholds, clinically meaningful impairment was identified in 31.6–47.6% of functional scales and 15.5–45.0% of symptom scales. **Conclusions:** Depressive symptom severity, functional limitations, and symptom burden were associated with poorer QoL among cancer patients. Integrating psychological and supportive care is essential to enhance well-being in Qassim.

## 1. Introduction

Cancer remains a major public health challenge worldwide and encompasses a broad range of diseases with substantial physical, psychological, and social consequences. In 2022, an estimated 20 million new cancer cases were reported globally [1,2]. In Saudi Arabia, the cancer burden is expected to double over the next two decades due to population ageing and the increasing prevalence of risk factors [3]. In the Qassim region, cancer incidence showed an increasing trend over 2002–2016, consistent with the broader rise in cancer burden reported in Saudi Arabia [4]. This trend has been attributed to rapid urbanization, lifestyle changes, obesity, and improved diagnostic practices [2,5,6]. As survival rates continue to improve due to advances in early detection and therapy, more individuals are now living with cancer as a chronic condition, which highlights the need to assess and address patients’ quality of life (QoL) and psychosocial well-being [7,8].

Quality of life is often considered a dynamic, multidimensional construct, encompassing all physical, psychological, social, and spiritual domains that reflect a patient’s overall functioning and well-being [9,10]. It not only provides insight into symptom burden and treatment tolerance but also serves as an independent prognostic indicator for overall survival in cancer patients [11,12,13].

Numerous factors are associated with the QoL of patients with cancer. A systematic review of 58 papers from 15 developing nations reported that older age, married status, spirituality, and engagement in palliative care were associated with higher QoL, whereas lower education and depression were associated with lower QoL [14]. Similarly, a large meta-analysis involving 94 interview-based studies found that approximately one-third of cancer patients experience depression, anxiety, or adjustment disorder, significantly impacting QoL [15]. Recent Saudi studies also confirm the substantial psychological burden in oncology populations; in a tertiary-care study in Riyadh, 46% of patients reported moderate-to-severe distress symptoms [3]. Such findings underscore the urgent need for integrating psychosocial assessment and supportive care into oncology practice, consistent with the Multinational Association of Supportive Care in Cancer (MASCC)’s perspective on comprehensive supportive care [8].

Regardless of other demographic factors, adolescent and young adult (AYA) cancer survivors were more likely to report “worse” or impaired QoL compared to the general population [16]. Decision-making in oncology requires balancing longevity and QoL, as patient preferences for length of life (LoL) versus QoL can vary with age, social support, and baseline functioning [17].

Given these considerations and the paucity of data from the Qassim region, the present study aims to explore the sociodemographic and psychosocial determinants of QoL among cancer patients. This study aimed to (1) assess health-related QoL among cancer patients in the Qassim region using the EORTC QLQ-C30, and (2) identify sociodemographic, clinical, and psychological factors associated with poor global QoL. This work seeks to contribute regional evidence to inform holistic cancer care models in Saudi Arabia that integrate physical, emotional, and social dimensions of patient well-being.

## 2. Materials and Methods

A cross-sectional study was conducted at the Oncology Centre, Qassim, Saudi Arabia, between January and June 2024. The study targeted adult cancer patients attending the center for treatment or follow-up care. Ethical approval was obtained from the Regional Research Ethics Committee, Qassim Institutional Review Board (Approval No. 607-45-1325). The study was conducted in accordance with the Declaration of Helsinki and applicable national and institutional research guidelines.

### 2.1. Study Objective and Outcome Definition

The primary objective of this study was to identify sociodemographic, clinical, and psychological factors associated with poor global QoL among cancer patients. The primary outcome variable was global QoL, dichotomized as poor (≤60) or good (>60) based on the EORTC QLQ-C30 global health status scale. Independent variables were grouped a priori into three domains: (1) sociodemographic factors, (2) clinical and treatment-related factors, and (3) psychological distress variables (anxiety and depression), in accordance with a biopsychosocial framework of cancer-related quality of life.

### 2.2. Study Population and Sampling

The target population included adult patients (aged ≥18 years) with a confirmed diagnosis of cancer, who were either undergoing treatment or attending follow-up appointments at the Oncology Centre. Patients were eligible if they could understand Arabic and provided informed consent. Only participants who completed the questionnaire were included in the final analysis. Exclusion criteria included patients with cognitive impairment, severe psychiatric illness, or terminal medical instability that could preclude meaningful participation. A consecutive sampling approach was used to recruit eligible participants during the study period.

The required sample size was estimated a priori using Cochran’s formula for prevalence studies. Assuming a low-quality-of-life prevalence among cancer patients of 82%, a 95% confidence level, and a 5% margin of error, the sample size was calculated to be 227 participants. Over the study period, 233 eligible patients were identified, of whom 187 completed the questionnaire, corresponding to a response rate of 80.3%. Accordingly, the final analysis included 187 patients who completed the questionnaire during the study period.

### 2.3. Data Collection and Survey Instrument

Data were collected through face-to-face structured interviews conducted by trained research staff. The survey instrument consisted of four main sections: (1) sociodemographic and clinical characteristics, (2) QoL assessment, (3) depression and anxiety screening, and (4) beliefs and coping behaviors. Sociodemographic information included age, gender, marital status, education level, employment status, nationality, and region of residence. Clinical variables included cancer type and stage, time since diagnosis, treatment modality, psychiatric history, and use of alternative therapies.

### 2.4. Quality of Life Assessment

QoL was assessed using the validated Arabic version of the European Organization for Research and Treatment of Cancer (EORTC; Brussels, Belgium) Quality of Life Questionnaire-C30 (QLQ-C30) [3,18]. This widely used instrument evaluates multiple dimensions of QoL among cancer patients and has been validated in multiple populations, including Arabic-speaking cohorts. It comprises five functional subscales (physical, role, cognitive, emotional, and social functioning), one global health status/QoL scale, and nine symptom subscales (fatigue, pain, nausea and vomiting, dyspnea, insomnia, appetite loss, constipation, diarrhea, and financial difficulties). Most items are rated on a 4-point Likert scale (1 = not at all, 4 = very much), except for the global QoL scale, which uses a 7-point response format.

Raw scores were linearly transformed to a 0–100 scale, where 0 represents the minimum possible score, and 100 represents the maximum possible score, according to the EORTC scoring guidelines [19,20]. Higher scores on functional and global QoL scales indicate better functioning or well-being, whereas higher scores on symptom scales indicate greater symptom severity [19,20]. The EORTC QLQ-C30 consists of 30 items. QoL was categorized into “poor” (≤60) and “good” (>60) based on cutoffs from Derogar et al. [3,18,21].

### 2.5. Assessment of Psychological Distress

Depression and anxiety were evaluated using the Arabic-language versions of the Patient Health Questionnaire-9 (PHQ-9) and Generalized Anxiety Disorder-7 (GAD-7), respectively [22,23]. Both tools have been validated for use in Arabic-speaking populations and have demonstrated high internal consistency [24,25]. In the current sample, both psychological scales showed good internal consistency, with Cronbach’s α values of 0.841 for the PHQ-9 and 0.878 for the GAD-7. The PHQ-9 consists of nine items, each scored from 0 to 3. The total score ranges from 0 to 27, with higher scores indicating greater severity of depressive symptoms. A total score ≥ 10 was used to indicate clinically significant depression. In addition, the algorithmic criteria for major depressive disorder were applied: a score of “more than half the days” on either item 1 or 2, combined with at least four other symptoms and a response of “several days” or more on item 9.

The GAD-7 assesses anxiety severity using seven items, each scored from 0 (not at all) to 3 (nearly every day). The total score ranges from 0 to 21, with higher scores indicating greater severity of anxiety symptoms. Scores were categorized as follows: 0–4 (none), 5–9 (mild), 10–14 (moderate), and 15–21 (severe). A score ≥ 10 was used as the threshold for clinically significant anxiety, consistent with the prior literature [22,23]. The PHQ-9 and GAD-7 were used in this study as screening measures of depressive and anxiety symptom severity and do not constitute formal diagnostic instruments.

### 2.6. Statistical Analysis

All statistical analyses were performed using IBM SPSS Statistics version 30 (IBM Corp., Armonk, NY, USA). Continuous variables were reported as means and standard deviations or medians and interquartile ranges, depending on their distribution. Categorical variables were summarized as frequencies and percentages. The Shapiro–Wilk test was used to assess normality for continuous variables. Associations between QoL categories and categorical variables (e.g., gender, marital status, cancer type) were analyzed using the chi-square test or Fisher’s exact test, as appropriate. Comparisons of non-normally distributed continuous variables between groups were performed using the Mann–Whitney U test. To identify factors associated with global QoL, multivariable binary logistic regression was conducted. The dependent variable was global QoL, coded as 0 = poor QoL and 1 = good QoL. The model included clinically relevant variables selected a priori according to the study objectives and the biopsychosocial framework, including sociodemographic, clinical, treatment-related, and psychological variables. PHQ-9 and GAD-7 scores were entered into the model as continuous variables. Model fit was assessed using the Hosmer–Lemeshow goodness-of-fit test, Cox–Snell R^2^ and Nagelkerke R^2^, and multicollinearity was evaluated using variance inflation factors; no evidence of problematic multicollinearity was identified. Given the exploratory nature of the study and the large number of comparisons conducted, the results of univariate analyses should be interpreted cautiously. The primary inferential analysis was based on the multivariable logistic regression model. Adjusted odds ratios (ORs) and 95% confidence intervals (CIs) were calculated. A *p*-value < 0.05 was considered statistically significant.

## 3. Results

A total of 233 cancer patients met the inclusion criteria, of whom 187 completed the questionnaire, corresponding to an 80.3% response rate. The median age of participants was 48 years (range, 18–94). The majority of participants were female (73.8%) and Saudi (84.5%), and most were married (81.3%). A significant portion had a high school education or lower (63.1%) and a monthly income of 10,000 SAR or less (67.4%). The median time since cancer diagnosis was 7 months (IQR: 4.00–14.25). The median anxiety score was 2 (IQR, 0–7) and the median depression score was 6 (IQR, 3–11). The mean global health status score was 73.8 (*SD* = 22.2). Most patients (*n* = 139; 74.3%) had good global QoL (score > 60), whereas 48 (25.7%) reported poor global QoL. No statistically significant associations were observed between sociodemographic factors and global QoL, although older participants and Saudi nationals tended to report better global QoL than their counterparts (see Table 1). The mean scores of physical functioning (*p* < 0.001), role functioning (*p* < 0.001), cognitive functioning (*p* < 0.001), and social functioning (*p* < 0.001) were significantly higher among patients with good global QoL. Conversely, symptom scales were significantly higher among patients with poor QoL, including fatigue (*p* < 0.001), nausea and vomiting (*p* = 0.005), pain (*p* < 0.001), dyspnea (*p* = 0.010), insomnia (*p* < 0.001), appetite loss (*p* = 0.022), constipation (*p* = 0.042), diarrhea (*p* = 0.008), and financial difficulties (*p* = 0.003) (see Table 2).

Table 3 summarizes participant clinical characteristics. Among 187 cancer patients, breast cancer was the most common (52.2%), followed by colorectal cancer (16.1%), gynecological cancer (8.3%), lymphoma (6.7%), and others (16.7%). Nearly one-half (48.7%) had an unknown cancer stage; Stage 2 (18.7%) and Stage 3 (12.8%) were the next most frequent. Chemotherapy history was reported by 84% of patients, and radiotherapy was reported by 28.9%. Most participants (92.0%) had no psychiatric history. The leading perceived cause of cancer was “evil eye or magic” (33.7%), and 56.7% used Islamic Ruqya as an alternative treatment. Regarding the association between clinical characteristics and global QoL, in the univariate analysis, hormonal therapy (*p* = 0.022) and psychiatric history were significantly associated with poor global QoL (visiting a psychiatrist, *p* = 0.011; diagnosed with a psychiatric disorder, *p* = 0.001; and taking psychiatric medication, *p* = 0.036). Moreover, other factors, such as cancer type, cancer stage, personal beliefs, and alternative treatments, were not significantly associated with global QoL. Furthermore, there were no significant differences in healthcare satisfaction or social support satisfaction between patients with poor global QoL and those with good global QoL. These univariate analyses were exploratory and were interpreted cautiously. The primary inferential analysis was based on the multivariable model that included clinically relevant variables selected a priori.

Using a cutoff score of ≥10 on the GAD-7, 12.8% (24/187) of participants were classified as having clinically significant anxiety. This was significantly associated with global QoL: anxiety was present in 33.3% of participants with poor global QoL, compared with 5.8% among those with good global QoL. Using a broader cutoff score of ≥5 on the GAD-7, mild to severe anxiety was observed in 34.2% (64/187) of participants, including 12.1% with moderate to severe anxiety. Among individuals with poor global QoL, 56.2% experienced anxiety (with 12.8% reporting moderate to severe anxiety). In contrast, 26.6% of patients with good QoL showed symptoms of anxiety (5.7% moderate to severe anxiety); see Table 4.

Depression, indicated by a PHQ-9 cutoff score of ≥10, was identified in 29.9% (56/187) of participants. This was significantly associated with global QoL: depression was present in 56.3% (27/48) of participants with poor global QoL, compared with 20.9% (29/139) of participants with good global QoL. Using the diagnostic algorithm approach (individuals who scored 2 or more “more than half the days” in Questions #1 or #2, plus four or more other questions, and a score of “several days” or more in Question #9 on the PHQ-9), 10.7% of participants were identified as having depression, which was also significantly associated with poor global QoL. Among participants with poor global QoL, 25.0% met the diagnostic algorithm criteria for depression, compared with 5.8% of those with good global QoL; see Table 4.

A multivariable logistic regression analysis was conducted to identify factors associated with global QoL among cancer patients. The dependent variable was coded as 0 = poor QoL and 1 = good QoL. The overall model demonstrated acceptable fit (Hosmer–Lemeshow χ^2^ = 2.613, df = 8, *p* = 0.956) and explained a moderate proportion of the variance in QoL (Cox–Snell R^2^ = 0.233; Nagelkerke R^2^ = 0.342). In the adjusted model, higher depression scores were significantly associated with lower odds of reporting good QoL (AOR = 0.85, 95% CI [0.77–0.95], *p* = 0.004). Anxiety score, age, sex, cancer type, treatment modalities, and psychiatrist visit were not statistically significant in the adjusted model (Table 5).

The cohort reported a mean global health status score of 73.8 (*SD* = 22.2). Among the five functional scales, cognitive functioning had the highest mean score (73.9, *SD* = 29.2), while role functioning had the lowest mean score (67.6, *SD* = 33.8). Regarding symptom burden, fatigue had the highest mean score (44.7, *SD* = 31.1), closely followed by insomnia (44.0, *SD* = 40.2). Conversely, the smallest mean scores were observed in diarrhea (16.9, *SD* = 27.1) and financial difficulties (18.0, *SD* = 32.8). In addition to the dichotomized global QoL outcome used in the regression analysis, the original continuous EORTC QLQ-C30 scores were also analyzed descriptively to provide a more comprehensive assessment of health-related quality of life (Table 6).

Analysis of the TCI thresholds for the functioning scales demonstrated that a substantial proportion of patients reported clinically relevant impairment across all domains. The percentage of patients scoring below the TCI cutoff (indicating a problem) ranged from 31.6% to 47.6%. Physical functioning (PF) was the most affected domain, with nearly one-half of the sample (47.6%, *n* = 89) scoring below the threshold (<83). This was followed by role functioning (RF) at 39.0% (*n* = 73) and emotional functioning (EF) at 34.8% (*n* = 65). The domains with the lowest reported impairment were social functioning (SF) (32.6%, *n* = 61) and cognitive functioning (CF; 31.6%, *n* = 59); see Table 7.

In analyzing symptom scale data using the Therapeutic Change Index (TCI) cutoff, which defines a clinically important problem, fatigue (FA) was the most prevalent issue, affecting 45.0% (*n* = 84) of cases (TCI cutoff > 39). Pain (PA) followed as the second most common problem, observed in 38.0% of cases (*n* = 71; TCI cutoff > 25). Other significant symptoms included sleep disturbances (SL) (32.6%, *n* = 61) and appetite loss (AP) (28.9%, *n* = 54), both with a TCI cutoff of >50. Symptoms like nausea and vomiting (NV) (25.7%, *n* = 48), constipation (CO) (25.1%, *n* = 47), and dyspnea (DY) (19.3%, *n* = 36) were also observed, while financial impact (FI) (17.6%, *n* = 33) and diarrhea (DI) (15.5%, *n* = 29) were the least prevalent; see Table 8.

## 4. Discussion

This study represents the first comprehensive assessment of health-related quality of life (HRQoL) among cancer patients in the Qassim region of Saudi Arabia, utilizing the validated Arabic version of EORTC QLQ-C30 [18,20]. Our findings establish a preliminary regional baseline and identify areas of clinical and research interest in this understudied population. The study was guided by a biopsychosocial framework, in which clinical disease factors, functional limitations, symptom burden, and psychological distress are considered relevant correlates of patients’ global QoL.

EORTC results can be interpreted by comparison with normative data, thresholds for clinical importance, minimal important differences, and the EORTC’s reference manual for pooled international cancer patient data [26,27,28,29,30]. Comparison with general population data reveals a picture of impaired function and greater symptom burden among cancer patients in Qassim. Across all functional scales, mean scores were consistently lower than established norms (e.g., physical functioning at 69.6 vs. a normative score of 85.1). Conversely, mean scores for all symptom scales were considerably higher, indicating substantial distress (e.g., fatigue 44.7 vs. normative 29.5; pain 36.4 vs. normative 23.5). The TCI offers a clinically meaningful estimate of the patient burden [27]. A large proportion of the Qassim cohort reported clinically significant problems across key functional and symptom scales. Specifically, 47.6% of patients scored below the TCI threshold for physical functioning (PF; <83), while 45.0% and 38.0% scored above the TCI threshold for fatigue (FA; >39) and pain (PA; >25), respectively. A recent global meta-analysis has placed the prevalence of pain after cancer treatment near 39% [31].

Compared to the international pooled cancer patient reference values, the Qassim cohort’s mean global QoL score of 73.8 (*SD* 22.2) was higher (better) than the international reference of 61.3 [30], yet accompanied by lower functional status—particularly in physical functioning (69.6 vs. 76.7), cognitive functioning (73.9 vs. 82.6), and social functioning (72.8 vs. 75.0)—and substantially greater symptom burden in fatigue (44.7 vs. 34.6), pain (36.4 vs. 27.0), insomnia (44.0 vs. 28.9), and appetite loss (33.0 vs. 21.1) [27]. This finding—where global QoL status is maintained despite specific symptom reports—is reported in QoL research and may indicate response shift, a concept in which patients change their internal standards for QoL after diagnosis and surviving severe negative experiences, or may reflect strong family and social support systems [32]. An intra-country comparison with a Riyadh cohort [3] revealed a similar proportion of patients reporting good global QoL (Qassim: 74.33% vs. Riyadh: 73.2%, using the same cutoff of global QoL score ≤ 60), but the Qassim group demonstrated lower functional status (physical functioning: 69.6 vs. 80.6; role functioning: 67.6 vs. 83.1; cognitive functioning: 73.9 vs. 82.9; social functioning: 72.8 vs. 81.2) and greater symptom severity (fatigue: 44.7 vs. 36.9; pain: 36.4 vs. 27.5). Both studies were limited by a high proportion of unknown disease stage (Qassim: 48.7%; Riyadh: 51.4%), necessitating a focus on the overall case mix for comparison. The high proportion of patients with unknown cancer stage is an important limitation, as illness severity affects QoL—for example, inpatients were reported to have lower scores (68.6) than outpatients (72.3), highlighting the impact of patient acuity [7].

Our results suggest an association between psychological distress and global QoL. Anxiety was present in 12–34% of participants and depression in 10–29%, depending on scoring criteria; both were associated with lower global QoL in the unadjusted analyses. Additionally, a psychiatric history, particularly psychiatrist visits, was associated with poorer global QoL in the unadjusted analysis. In multivariable analysis, however, the depression score was the only variable independently associated with QoL, whereas the anxiety score, psychiatrist visit, and selected clinical factors were not statistically significant after adjustment. Another study, which was done in Riyadh, Saudi Arabia, reported a 21.7% and 29% anxiety and depression prevalence, respectively, using the same cutoff score used in this study [3]. This variation in prevalence may be related to differences in study timing, geographic location, lower functional status, higher symptom burden, cancer type, and sample characteristics. A systematic review and meta-analysis conducted in February 2025 revealed that the global prevalence of depression and anxiety in cancer patients was 33.16% (95% CI = 27.59–38.74) and 30.55% (95% CI = 24.04–37.06) [31]. The observed associations between QoL and symptoms of anxiety and depression are supported in the literature. For example, a longitudinal study from Greece showed that psychological distress symptoms, including depression and anxiety, were significantly associated with lower HRQoL among early non-metastatic colorectal cancer patients [33]. Moreover, a local study showed that depression, anxiety, and distress were significantly associated with poor global QoL [3].

Patients’ beliefs about cancer etiology and treatment were comparable between this study and the Riyadh cohort [3]. “Evil eye or magic” was the most common perceived cause in Qassim (33.7%) and Riyadh (28.6%), and Islamic Ruqya was used as an alternative treatment by 56.7% and 48.6% of patients, respectively [3]. While these beliefs did not demonstrate a statistically significant association with global QoL in this study, their high prevalence highlights the cultural and spiritual framework through which patients in Qassim understand and cope with their illness.

These findings have important implications for policy and practice. They support the incorporation of routine HRQoL and psychological distress screening into oncology care and highlight the need for multidisciplinary supportive services that address symptom burden, psychological well-being, and functional limitations. They also support a biopsychosocial understanding of cancer care, in which QoL is shaped by interacting clinical, psychological, and functional factors.

The primary strength of this study is its originality, as it is the first to focus on HRQoL in Qassim and to use validated, Arabic-translated instruments (EORTC QLQ-C30, GAD-7, PHQ-9). Nevertheless, the study has certain limitations. The cross-sectional design precludes the determination of causal relationships. Furthermore, the single-center recruitment may limit the generalizability of findings beyond the Qassim Health Cluster. Also, a large proportion of participants had an unknown cancer stage (48.7%), which limited the assessment of the relationship between disease stage and HRQoL and may have influenced the interpretation of the findings. Moreover, only 15 participants reported a previous diagnosis of a psychiatric disorder or psychotropic treatment. This low number likely stems from the core question, “Have you ever visited a psychiatrist?”, which may have led participants to omit subsequent related questions if they answered negatively. Furthermore, the question’s phrasing may not have captured instances in which a psychiatrist saw a patient during an inpatient consultation. This design is problematic because non-psychiatrist clinicians (e.g., family medicine physicians) often provide psychiatric diagnosis and intervention. Future research should use broader instruments to capture psychiatric history more comprehensively.

## 5. Conclusions

This study found that overall QoL was relatively preserved despite a significant burden from severe physical symptoms (fatigue and pain) and impaired functional status in Qassim. Psychological distress, including anxiety and depression, was common and associated with poorer QoL. These findings support a biopsychosocial approach to cancer care. We recommend integrating mental health screening and multidisciplinary supportive care protocols into oncology care to address psychological distress, symptom burden, and functional limitations and improve patient well-being. Future longitudinal studies are needed to clarify temporal relationships.

## Figures and Tables

**Table 1:** Sociodemographic characteristics of the patients and their global quality of life (N = 187).

Characteristics	Global Health Status (QoL)			
	Overall	Good	Poor	*p*-Value
	*N* = 187			
Age group	*n* (%)	*n* (%)	*n* (%)	0.579
18–42	51 (27.3)	36 (25.9)	15 (31.3)	
43–50	50 (26.7)	36 (25.9)	14 (29.2)	
>50	86 (46.0)	67 (48.2)	19 (39.6)	
Sex				0.173
Female	138 (73.8)	99 (71.2)	39 (81.3)	
Male	49 (26.2)	40 (28.8)	9 (18.8)	
Nationality				0.258
Saudi	158 (84.5)	115 (82.7)	43 (89.6)	
Non-Saudi	29 (15.5)	24 (17.3)	5 (10.4)	
Marital status				0.237
Widow	10 (5.3)	6 (4.3)	4 (8.3)	
Single	17 (9.1)	12 (8.6)	5 (10.4)	
Married	152 (81.3)	117 (84.2)	35 (72.9)	
Divorced	8 (4.3)	4 (2.9)	4 (8.3)	
Education Level				0.427
Higher school or below	118 (63.1)	90 (64.7)	28 (58.3)	
Diploma or bachelor’s or higher	69 (36.9)	49 (35.3)	20 (41.7)	
Monthly income (SAR)				0.233
<10,000	126 (67.4)	97 (69.8)	29 (60.4)	
≥10,000	61(32.6)	42 (30.2)	19 (39.6)	
Number of family members				0.863
1–3 members	33 (17.6)	25 (18.0)	8 (16.7)	
4–6	65 (34.8)	47 (33.8)	18 (37.5)	
7–9	59 (31.6)	43 (30.9)	16 (33.3)	
≥10	30 (16.0)	24 (17.3)	6 (12.5)	

**Table 2:** Relationship between global QoL, functional and symptom scales in cancer patients.

Quality of Life Domains	Overall (*N* = 187)	Poor	Good	
(EORTC QLQ-C30)		(*N* = 48)	(*N* = 139)	*p*-Value *
Functional scales	Mean (*SD*) Scale (100)			
Physical functioning	69.6 (24.1)	54.02 (25.6)	74.96 (21.2)	<0.001
Role functioning	67.6 (33.8)	53.5 (36.7)	72.5 (31.5)	<0.001
Emotional functioning	71.6 (30.8)	51.0 (32.8)	78.7 (26.7)	<0.001
Cognitive functioning	73.9 (29.2)	56.6 (29.9)	79.9 (26.5)	<0.001
Social functioning	72.8 (31.1)	57.6 (34.0)	78.1 (28.3)	<0.001
Symptom scales				
Fatigue	44.68 (31.1)	65.7 (27.4)	37.4 (28.9)	<0.001
Nausea and vomiting	20.41 (26.67)	29.16 (30.1)	17.38 (24.8)	0.005
Pain	36.36 (31.58)	56.25 (33.9)	29.49 (27.6)	<0.001
Dyspnea	19.25 (28.88)	29.16 (34.1)	15.82 (26.1)	0.010
Insomnia	44.03 (40.21)	61.80 (40.1)	37.88 (38.5)	<0.001
Appetite loss	32.98 (35.91)	44.44 (40.3)	29.01 (33.5)	0.022
Constipation	29.77 (36.73)	38.88 (39.7)	26.61 (35.2)	0.042
Diarrhea	16.93 (27.07)	26.38 (32.2)	13.66 (24.3)	0.008
Financial difficulties	18.00 (32.83)	29.86 (39.0)	13.90 (29.4)	0.003

* *p*-value for independent samples Mann–Whitney U.

**Table 3:** Clinical characteristics of the patients with cancer and their association with global quality of life (N = 187).

	Overall	Good	Poor	*p*-Value
	*n* (%)	*n* (%)	*n* (%)	
Type of cancer				
Breast	94 (52.2)	67 (49.6)	27 (60.0)	0.186
Colorectal cancer	29 (16.1)	19 (14.1)	10 (22.2)	
Gynecological cancer	15 (8.3)	13 (9.6)	2 (13.3)	
Lymphoma	12 (6.7)	11 (8.1)	1 (2.2)	
Others	30 (16.7)	25 (18.5)	5 (11.1)	
Time since cancer diagnosis				0.938
≤6 months	85 (45.7)	64 (46.4)	21 (43.8)	
>6 to ≤12 months	50 (26.9)	37 (26.8)	13 (27.1)	
>12 months	51 (27.4)	37 (26.8)	14 (29.2)	
Stage of cancer				
Stage 1	18 (9.6)	17 (12.2)	1 (2.1)	0.264
Stage 2	35 (18.7)	26 (18.7)	9 (18.8)	
Stage 3	24 (12.8)	17 (12.2)	7 (14.6)	
Stage 4	19 (10.2)	12 (8.6)	7 (14.6)	
Do not know	91 (48.7)	67 (48.2)	24 (50.0)	
Type of intervention ^+^				
Chemotherapy	157 (84.0)	118 (84.9)	39 (81.3)	0.533
Radiotherapy	54 (28.9)	41 (29.5)	13 (27.1)	0.750
Surgery	82 (43.9)	61 (43.9)	21 (43.8)	0.987
Hormonal therapy	34 (18.2)	20 (14.4)	14 (29.2)	0.022
Others	10 (5.3)	8 (5.8)	2 (4.2)	0.673
Psychiatric history				
Visited psychiatrist				0.011
No	172 (92.0)	132 (95.0)	40 (83.3)	
Yes	15 (8.0)	7 (5.0)	8 (16.7)	
Diagnosed with a psychiatric disorder				0.001 ^a^
No	8 (4.3)	6 (4.3)	2 (4.2)	
Yes	7 (3.7)	1 (0.7)	6 (12.5)	
Taking psychiatric medication				0.036 ^a^
No	8 (4.3)	4 (2.9)	4 (8.3)	
Yes	7 (3.7)	3 (2.2)	4 (8.3)	
Personal beliefs				
Perceived reason for having cancer				0.631
Evil eye or magic	63 (33.7)	49 (35.3)	14 (29.2)	
Genetics	18 (9.6)	14 (10.1)	4 (8.3)	
Health condition	49 (26.2)	38 (27.3)	11 (22.9)	
Other	17 (9.1)	11 (7.9)	6 (12.5)	
Unknown	40 (21.4)	27 (19.4)	13 (27.1)	
Use of another method to treat the disease				1.000 ^a^
Complementary medicine	12 (6.4)	9 (6.5)	3 (6.3)	
Special diet	4 (2.1)	3 (2.2)	1 (2.1)	
Islamic Ruqya	106 (56.7)	79 (56.8)	27 (56.3)	
No	65 (34.8)	48 (34.5)	17 (35.4)	
Measuring satisfaction with medical care and social support				
Mean (*SD*)				
	Overall	Good QoL	Poor QoL	
	Mean (*SD*)	Mean (*SD*)	Mean (*SD*)	*p*-value *
Healthcare	9.38 (1.4)	9.35 (1.46)	9.48 (0.99)	0.556
Social support	9.35 (1.4)	9.47 (1.24)	9.00 (1.71)	0.087

^+^ multiple responses. * *p*-value for an independent samples *t*-test. ^a^ Fisher’s Exact Test and others are chi-square tests.

**Table 4:** Association between anxiety and depression categories and global quality of life in cancer patients (N = 187).

	Overall	Poor	Good	
Variables	(*N* = 187)	(*N* = 48)	(*N* = 139)	*p*-Value
Anxiety				
None (score 0–4)	123 (65.8)	21 (43.8)	102 (73.4)	<0.001
Mild (score 5–9)	40 (21.4)	11 (22.9)	29 (20.9)	
Moderate (score 10–14)	17 (9.1)	11 (22.9)	6 (4.3)	
Severe (score 15–21)	7 (3.7)	5 (10.4)	2 (1.4)	
Depression (diagnostic algorithm approach)				
Not depressed	167 (89.3)	36 (75.0)	131 (94.2)	<0.001
Depressed	20 (10.7)	12 (25.0)	8 (5.8)	
Anxiety				
Not anxious (score < 10)	163 (87.2)	32 (66.7)	131 (94.3)	<0.001
Anxious (score ≥ 10)	24 (12.8)	16 (33.3)	8 (5.8)	
Depressed (scoring approach)				
Not depressed (score < 10)	131 (70.1)	21 (43.8)	110 (79.1)	<0.001
Depressed (score ≥ 10)	56 (29.9)	27 (56.3)	29 (20.9)	

**Table 5:** Univariate and multivariable logistic regression analysis of factors associated with global quality of life in cancer patients.

Variables	Crude OR	*p*-Value	Adjusted OR [95% CI]	*p*-Value
	[95% CI]			
Age group				
18–42	Ref		Ref	
43–50	0.68 [0.31–1.50]	0.339	0.63 [0.23–1.72]	0.366
>50	0.73 [0.33–1.62]	0.439	0.54 [0.20–1.42]	0.208
Sex				
Female	Ref		Ref	
Male	0.57 [0.25–1.29]	0.177	0.98 [0.30–3.16]	0.971
Cancer type				
Breast cancer	Ref		Ref	
Colorectal cancer	0.54 [0.24–1.23]	0.144	0.65 [0.22–1.93]	0.436
Lymphoma	0.36 [0.13–1.01]	0.053	0.28 [0.08–1.04]	0.057
Other cancers	2.67 [0.31–22.94]	0.372	6.63 [0.45–98.71]	0.170
Treatment modality				
Chemotherapy	0.77 [0.33–1.82]	0.554	0.54 [0.15–1.92]	0.339
Radiotherapy	0.89 [0.43–1.85]	0.751	0.44 [0.14–1.43]	0.174
Surgery	1.00 [0.51–1.93]	0.987	1.24 [0.47–3.32]	0.663
Hormone therapy	2.45 [1.12–5.36]	0.025	1.73 [0.53–5.62]	0.360
Other treatment	0.71 [0.15–3.48]	0.674	0.50 [0.05–4.60]	0.541
Psychological factors				
Anxiety score (GAD-7)	0.83 [0.77–0.90]	<0.001	0.95 [0.84–1.07]	0.370
Depression score (PHQ-9)	0.85 [0.80–0.91]	<0.001	0.85 [0.77–0.95]	0.004
Visited psychiatrist (Yes vs. No)	0.27 [0.09–0.78]	0.016	0.62 [0.16–2.46]	0.497

OR = odds ratio; CI = confidence interval. For continuous variables (PHQ-9 and GAD-7), odds ratios represent the change in odds associated with a one-unit increase in score.

**Table 6:** Descriptive statistics of health-related quality of life (HRQoL) across EORTC QLQ-C30 functional and symptom scales (N = 187).

EORTC QLQ-C30 Scale	Mean Score	Standard Deviation (*SD*)
Functional Scales		
Physical functioning	69.6	24.1
Role functioning	67.6	33.8
Emotional functioning	71.6	30.8
Cognitive functioning	73.9	29.2
Social functioning	72.8	31.1
Symptom Scales		
Fatigue	44.7	31.1
Pain	36.4	31.6
Nausea and vomiting	20.4	26.7
Insomnia	44.0	40.2
Dyspnea	19.3	28.9
Appetite loss	33.0	35.9
Constipation	29.8	36.7
Diarrhea	16.9	27.1
Financial difficulties	18.0	32.8
Global health status	73.8	22.2

**Table 7:** Therapeutic Change Index (TCI) thresholds and prevalence of clinically important impairment on EORTC QLQ-C30 functional scales.

Scale	TCI Cutoff	Clinically Important Cases (*n*)	Percentage (%)
Physical functioning	<83	89	47.6
Role functioning	<58	73	39.0
Emotional functioning	<71	65	34.8
Cognitive functioning	<75	59	31.6
Social functioning	<58	61	32.6

**Table 8:** Therapeutic Change Index (TCI) thresholds and prevalence of clinically important symptom burden on EORTC QLQ-C30 symptom scales.

Scale	TCI Cutoff	Clinically Important Cases (*n*)	Percentage (%)
Fatigue	>39	84	45.0
Pain	>25	71	38.0
Nausea and vomiting	>8	48	25.7
Sleep disturbances	>50	61	32.6
Dyspnea	>17	36	19.3
Appetite loss	>50	54	28.9
Constipation	>50	47	25.1
Diarrhea	>17	29	15.5
Financial impact	>17	33	17.6

## Data Availability

The data presented in this study are available on request from the corresponding author, the data are not publicly available due to privacy or ethical restrictions.

## Abbreviations

The following abbreviations are used in this manuscript:

AYA	Adolescent and young adult
CI	Confidence interval
EORTC QLQ-C30	European Organization for Research and Treatment of Cancer Quality of Life Questionnaire-C30
GAD-7	Generalized Anxiety Disorder-7
HRQoL	Health-related quality of life
IQR	Interquartile range
MASCC	Multinational Association of Supportive Care in Cancer
OR	Odds ratio
PHQ-9	Patient Health Questionnaire-9
QoL	Quality of life
SAR	Saudi Riyal
SD	Standard deviation
SPSS	Statistical Package for the Social Sciences
TCI	Therapeutic Change Index
